# Paternal education status significantly influences infants’ measles vaccination uptake, independent of maternal education status

**DOI:** 10.1186/1471-2458-12-336

**Published:** 2012-05-08

**Authors:** Anu Rammohan, Niyi Awofeso, Renae C Fernandez

**Affiliations:** 1 School of Business, Discipline of Economics, University of Western Australia, 35 Stirling Highway, Crawley, WA 6009, Australia; 2 School of Population Health, University of Western Australia, 35 Stirling Highway, Crawley, WA 6009, Australia; 3 School of Public Health and Community Medicine, University of New South Wales, Anzac Pde, Kensington, NSW 2052, Australia

## Abstract

****Background**:**

Despite increased funding of measles vaccination programs by national governments and international aid agencies, structural factors encumber attainment of childhood measles immunisation to levels which may guarantee herd immunity. One of such factors is parental education status. Research on the links between parental education and vaccination has typically focused on the influence of maternal education status. This study aims to demonstrate the independent influence of paternal education status on measles immunisation.

****Methods**:**

Comparable nationally representative survey data were obtained from six countries with the highest numbers of children missing the measles vaccine in 2008. Logistic regression analysis was applied to examine the influence of paternal education on uptake of the first dose of measles vaccination, independent of maternal education, whilst controlling for confounding factors such as respondent’s age, urban/rural residence, province/state of residence, religion, wealth and occupation.

****Results**:**

The results of the analysis show that even if a mother is illiterate, having a father with an education of Secondary (high school) schooling and above is statistically significant and positively correlated with the likelihood of a child being vaccinated for measles, in the six countries analysed. Paternal education of secondary or higher level was significantly and independently correlated with measles immunisation uptake after controlling for all potential confounders.

****Conclusions**:**

The influence of paternal education status on measles immunisation uptake was investigated and found to be statistically significant in six nations with the biggest gaps in measles immunisation coverage in 2008. This study underscores the imperative of utilising both maternal and paternal education as screening variables to identify children at risk of missing measles vaccination prospectively.

## **Background**

Measles is a highly contagious, yet vaccine preventable, viral infection, with a case fatality rate of up to 10%. Globally, an estimated 10 million cases and 164,000 deaths from measles occurred in 2008. Measles vaccination is a cost-effective intervention for averting measles infection. Vaccination for measles has been part of the World Health Organization’s (WHO) recommended immunization series since the inception of Expanded Programme on Immunization (EPI) in 1974. Routine measles vaccination coverage was selected as the third indicator of progress towards the Millennium Development Goal (MDG 4), of reducing under-five mortality rates by two-thirds by 2015, compared with 1990 baseline data.

According to the WHO, by 2008, measles vaccination coverage was 83% globally among children aged 12–23 months old. Nearly 700 million children aged between 9 months to 14 years living in high-risk countries, were vaccinated against the disease between 2000 to 2008, and globally measles deaths declined by 78% during this period, indicating that the global measles vaccination campaign successfully averted over 3.6 million deaths [1-3]. The lowest rates were in the South-East Asian (75%) and African (73%) regions. In low-income countries, 76% of children aged 12–23 months had received measles vaccination [3].

However, measles continues to be a major public health problem among children in developing nations, with measles deaths predominantly occurring among children aged below five years. Children living in developing countries with low income and poor health infrastructure are at the highest risk of measles-related morbidity and mortality. Most of the world’s measles-related deaths occur in nations with the lowest measles vaccine uptake [4,5].

In 2008 two-thirds of the 22.7 million children who missed receiving measles vaccine in lived in India, Nigeria, China, Democratic Republic of Congo, Pakistan, Ethiopia and Indonesia [4]. In India although the measles vaccination uptake increased to 70% in 2008, measles mortality is high, with about 300 children estimated to be dying from measles-related complications every day.

The major factors influencing measles and other childhood vaccinations operate at five different levels; (1) Intra-personal – Individual child’s characteristics; (2) Inter-personal – parental and household factors; (3) Community – community characteristics and service delivery factors; (4) Institutional: international coordination of vaccination efforts (5) Public policy – quality, coverage and enforcement of policies [6]. This study focuses on the interpersonal factors influencing vaccination, Our findings have implications for the other four levels which influence childhood vaccination.

Variables such as maternal education and occupation and household’s economic status, have consistently been shown to influence measles vaccination at an interpersonal level in developing countries [7-9]. However, much of this research has focused on the role of maternal education status, despite the patriarchal nature of most nations with low measles vaccination uptake. The few studies on the impact of paternal education on vaccination uptake were largely based on small samples in limited settings. For example, a study based on a 2006 survey of slum children in Bangladesh showed that paternal occupation significantly influenced vaccination uptake, with children with fathers in a business or service occupation being 1.059 times and 1.107 times more likely to vaccinate their children, relative to children whose fathers were labourers [9]. A recent study from Pakistan found that while maternal education status was influential in improving child nutritional outcomes, father’s education was a more important factor in one-off type health decisions such as receiving vaccination, in comparison to day-today decisions related to nutrition [10].

A study based on data from the 1992 Indian National Family and Health Survey found that, in rural areas, children with literate fathers were more likely to be vaccinated, even if the mothers are illiterate [11]. The positive influence of high paternal education status on measles vaccination uptake in developed nations was also demonstrated in a state-wide study of 1003 children aged 2 in Ohio, United States [12].

This paper analyses the probability of a child being vaccinated against measles, specifically focusing on the influence of paternal education status at different levels of maternal education. The key variable of interest is the likelihood that a child aged under 5 years has been vaccinated for measles.

## **Methods**

This analysis focused on six countries with the lowest levels of measles vaccination coverage for children in 2008 - Indonesia, India, Pakistan, Nigeria, Democratic Republic of Congo and Ethiopia. For these countries, comparable data was accessed from the Demographic Health Survey (DHS); the 2007 *Indonesia Demographic and Health Survey* (IDHS), the Indian *National Family Health Survey* (NFHS) 2005–06, the 2006–07 *Pakistan Demographic Health Survey* (PDHS), the 2008 *Nigeria National Demographic and Health Survey* (NDHS), the 2007 *Congo Democratic Republic Demographic Health Survey* (EDS-RDC) and the 2005 *Ethiopia Demographic and Health Survey* (EDHS). These datasets are produced by ORC Macro for the Measure DHS (Demographic and Health Surveys) Project, which is funded by the US Agency for International Development (USAID). Measure DHS distributes survey data files at no charge for legitimate academic research.

The Demographic and Health Survey was considered the best available gold standard for comparison of immunisation data. Investigations of the quality of DHS methodologies concluded that it is nationally representative and relatively free of systematic bias [13].

Table 1 describes the variables used in the analysis. maternal and paternal education were included as categorical variables indicating the highest level of education attained by the child’s mother and father, that is, no education (reference category), primary, secondary or higher education.

**Table 1:** Variable definitions and sociodemographic characteristics of the sample

	India	Nigeria	Congo	Ethiopia	Pakistan	Indonesia
**Variable**	%	%	%	%	%	%
Child received measles vaccination	0.55	0.37	0.58	0.28	0.52	0.73
Child’s sex- male	0.52	0.51	0.49	0.50	0.52	0.52
Father’s education- none	0.23	0.40	0.10	0.59	0.37	0.03
Father’s education- primary school	0.15	0.22	0.26	0.27	0.16	0.40
Father’s education- above Secondary schooling	0.62	0.38	0.64	0.14	0.47	0.57
Mother’s education-No education	0.39	0.50	0.24	0.38	0.67	0.04
Mother’s education- Primary school	0.14	0.23	0.42	0.36	0.14	0.40
Mother’s education: above Secondary schooling	0.46	0.27	0.34	0.26	0.19	0.56
Mother’s occupation: agriculture	0.21	0.20	0.51	0.16	0.11	0.22
Mother’s occupation : unskilled		0.001	0.02	0.01	0.03	0.40
Mother’s occupation: skilled	0.13	0.49	0.23	0.11	0.14	0.29
Mother not working	0.66	0.31	0.24	0.72	0.72	0.49
urban	0.39	0.45	0.40	0.13	0.35	0.38
Wealth quintile- poorest	0.17	0.26	0.22	0.25	0.23	0.30
Wealth quintile- poorer	0.18	0.23	0.21	0.19	0.21	0.20
Wealth quintile- middle	0.21	0.19	0.19	0.19	0.20	0.17
Wealth quintile- Rich	0.22	0.17	0.21	0.17	0.19	0.17
Wealth quintile- Richest	0.22	0.15	0.17	0.20	0.17	0.16
**Sample size**	45462	22344	7431	8261	8268	15065

Maternal and paternal occupation variables were included to further investigate differences in immunisation coverage across socio-economic groups. Occupation variables were defined as ‘not working’ (reference category), ‘agricultural self-employed’, ‘manual labour’ and ‘professional/clerical/services’. Household economic status is measured using the DHS wealth index quintiles, ranging from poorest (reference category) to richest.

Logistic regression analysis was used to explore the likelihood of a child under five years of age being vaccinated for measles, separately for each country. The study focused on the role of paternal education within each level of maternal education, while simultaneously quantifying the effects of multiple characteristics. The analysis controlled for respondent’s age, urban or rural residence, province/state of residence, religion, wealth quintile and partner’s occupation.

## **Results**

The pattern of measles vaccination coverage was examined descriptively for increasing levels of paternal education, within each level of maternal education (Table 2).

**Table 2:** Measles vaccination coverage by paternal education, within maternal education groups

**Parental education levels**		**Measles vaccination**											
**Maternal highest education level**	**Partner’s education level**	**India**		**Nigeria**		**Pakistan**		**Ethiopia**		**Congo**		**Indonesia**	
		**No**	**Yes**	**No**	**Yes**	**No**	**Yes**	**No**	**Yes**	**No**	**Yes**	**No**	**Yes**
**No Education**	**No Education**	5,69	2884	6,70	1,29	1660	1092	3820	1023	302	214	154	89
		(66.1%)	(33.9%)	(84.2%)	(15.8%)	(60.3%)	(39.7%)	(78.9%)	(21.1%)	(58.5%)	(41.5%)	(63.4%)	(36.6%)
	Primary***	1,95	1,38	1,27	410	539	449	1176	411	376	333	176	138
		(58.7%)	(41.3%)	(75.6%)	(24.4%)	(54.6%)	(45.5%)	(74.1%)	(25.9%)	(53.0%)	(47.0%)	(56.1%)	(43.9%)
	**Secondary or Higher**	3,38	2,64	1,01	440	912	954	238	147	299	263	46	48
		(56.1%)	(43.9%)	(69.8%)	(30.2%)	(48.9%)	(51.1%)	(61.8%)	(38.2%)	(53.2%)	(46.8%)	(48.9%)	51.1%
**Primary**	**No Education**	619	591	568	246	108	119	248	117	88	118	112	119
		(51.2%)	(48.8%)	(69.8%)	(30.2%)	(47.6%)	(52.4%)	(68.0%)	(32.1%)	(42.7%)	(57.3%)	48.5%	51.5%
	**Primary**	(48.7	(51.3	(53.7	(46.4	(45.3	(54.7	(66.3	(33.7	(49.1	(50.9	37.7	62.3
		%)	%)	%)	%)	%)	%)	%)	%)	%)	%)	%	%
	**Secondary or Higher**	1,59	2,02	1,10	1,03	259	428	220	180	820	1,03	593	1496
		(44.1%)	(55.9%)	(51.6%)	(48.4%)	(37.7%)	(62.3%)	(55.0%)	(45.0%)	(44.2%)	(55.8%)	(28.4%)	(71.6%)
**Secondary or Higher**	**No Education**	428	514	114	77	30	69	10	13	16	22	11	27
		(37.4%)	(54.6%)	(59.7%)	(40.3%)	(30.3%)	(69.7%)	(43.48%)	(56.52%)	(42.1%)	(57.9%)	(28.9%)	(71.1%)
	**Primary**	584	979	392	606	55	81	38	64	48	98	378	1019
		(37.4%)	(62.6%)	(39.3%)	(60.7%)	(40.4%)	(59.6%)	(37.25%)	(62.75%)	(32.9%)	(67.1%)	(27.1%)	(72.9%)
	**Secondary or Higher**	5,56	13,0	1,82	3,11	380	1016	180	346	688	1,64	1358	6897
		(30.0%)	(70.1%)	(36.9%)	(63.1%)	(27.2%)	(72.8%)	(34.22%)	(65.78%)	(29.5%)	(70.6%)	(16.5%)	(83.5%)

Note: Within levels of maternal education, differences in measles vaccination coverage by paternal education level are significantly different at the **1% level.

In India when both parents had no education, the proportion of children who were vaccinated for measles was 33.9%, and this increased to 43.9% even when the mother had no education, but the father had Secondary schooling and above. Similarly, for Indonesia when both parents had primary schooling, 55.1% of children were vaccinated, increasing to 75.6% when the mother had primary schooling, but father’s education was Secondary schooling and above. For Nigeria, when both parents had no education, 15.8% of children were vaccinated, and this figure nearly doubled when the father had secondary schooling, but the mother had no education.

In Table 2 the significance of differences in measles vaccination coverage between paternal education categories is tested, while controlling for maternal education level.

A striking finding was that, in all six countries studied, the proportion of children who were vaccinated for measles increased as father’s education increased, regardless of mother’s education levels. When both parents had no education, children were significantly less likely to be immunised against measles (p-value < 0.05).

The most conclusive results were under the following two scenarios. First, when maternal education is primary or secondary schooling, the proportion of children receiving measles immunisation was significantly higher when paternal education is secondary or higher (p-value < 0.05). Second, when maternal education was primary and paternal education is no education or primary, the proportion of children *not* receiving measles vaccination was significantly higher (p-value < 0.05). This was also seen when maternal education level was secondary and paternal education as primary (p-value < 0.05). These findings confirm paternal education status as an independent correlate of measles immunisation coverage.

To investigate the combined influence of these factors, the change in measles vaccination coverage with increasing paternal education within a particular maternal education level was analysed. The correlation between maternal and paternal education levels and measles vaccination was established in the logistic regression analysis. Table 3 presents the results of a logistic regression analysis. These results show the estimated likelihood of the child being vaccinated for measles separately for each country and for disaggregated samples for different levels of maternal education. Specifically, we estimated a series of logistic regression models for each of our six countries, separately for the full sample; sample where the mothers had no schooling; sample where the mothers had only primary schooling; and sample where the mothers had secondary and higher levels of schooling. Variables including household wealth, rural/urban residence, dummy variables for province of residence, occupations of mother and father and child’s religion were included in all regressions.

**Table 3:** Logistic regression results

	**Full sample**	**Mother has no education**	**Mother has primary education**	**Mother has secondary or higher education**
**Father with primary education**				
Congo	0.238* (0.127)	0.408***(0.156)	−0.099 (0.201)	0.465 (0.533)
	N = 7431	N = 1780	N = 3139	N = 2514
Ethiopia	0.133 (0.087)	0.160 (0.098)	−0.132 (0.205)	0.567 (0.660)
	N = 8261	N = 6395	N = 1267	N = 585
Nigeria	0.380***(0.076)	0.272***(0.104)	0.375***(0.121)	0.751*** (0.247)
	N = 22344	N = 11147	N = 5072	N = 6125
India	0.194*** (0.047)	0.198***(0.056)	0.039 (0.106)	0.303** (0.127)
	N = 45462	N = 17871	N = 21077	N = 6514
Pakistan	0.1384 (0.096)	0.1908* (0.104)	0.3198 (0.269)	−0.9583** (0.477)
	N = 8268	N = 5514	N = 1149	N = 1605
Indonesia	0.231** (0.112)	0.191 (0.209)	0.321** (0.154)	−0.014 (0.403)
	N = 14264	N = 622	N = 5693	N = 7949
**Father with second or higher education**				
Congo	0.251* (0.132)	0.396** (0.185)	−0.095 (0.210)	0.526 (0.475)
Ethiopia	0.433*** (0.130)	0.594***(0.167)	0.150(0.248)	0.351(0.652)
Nigeria	0.472*** (0.076)	0.441*** (0.101)	0.473***(0.120)	0.807*** (0.236)
India	0.324*** (0.042)	0.334***(0.050)	0.189*(0.100)	0.400*** (0.111)
Pakistan	0.446*** (0.083)	0.393***(0.091)	0.6756***(0.220)	0.0180 (0.391)
Indonesia	0.550***(0.117)	0.494*(0.288)	0.670***(0.161)	0.255(0.400)

Notes: Table displays coefficients and robust standard errors in parentheses. *p < 0.1, **p < 0.05, ***p < 0.01 refer to levels of significance at 10%, 5% and 1% levels respectively. The same set of controls were used for each country (religion, mother’s occupation dummies, father’s occupation dummies, wealth quintile dummies, rural residence and province dummy variables).

Table 3, summarises the coefficients for paternal education within different levels of maternal education. The coefficients presented in Table 3 are for the influence of father’s education

After controlling for the influence of all other potentially confounding variables, when considered separately, a significant finding of this study is the robust statistically significant finding of the important role of father’s education in influencing the likelihood of a child’s measles vaccination. For the six countries analysed in the sample, there is a large, statistically significant, and positive correlation between measles vaccination and father having secondary or higher levels of education, within each level of maternal education. It is noteworthy, that holding all other factors constant in all the six countries, even when the mother has no education, having a father with secondary schooling or higher levels of education is significantly and positively correlated with a higher likelihood of the child being vaccinated for measles (p-value <0.01).

## **Discussion**

In this study we investigated the role of paternal education in determining the likelihood that children receive measles vaccination in six countries with the lowest rates of vaccination coverage. The likelihood that a child received measles vaccination increased with increasing levels of paternal education. This result was shown to be independent of maternal education levels. The positive correlation between paternal education levels and measles vaccination remained significant after controlling for a number of possible confounders.

The results of the current study comply with previous studies investigating factors associated with childhood vaccination coverage in both developing and developed countries. A study conducted in Istanbul found that the rate of non-vaccination was 2.3 times higher among children whose fathers had less than secondary education compared to those whose fathers had attained secondary education or higher [14]. Marks et al. investigated vaccination in the United States, finding that paternal education was a significant predictor of childhood vaccination coverage. This result was found to be independent of maternal education levels [12]. Breierova and Duflo showed that paternal and maternal education levels were equally important determinants of child mortality, and that this may reflect “assortative matching”, i.e., maternal and paternal education levels are correlated because more educated people are likely to choose more educated spouses [15]. Ours is the first nationally representative study to explore the impact on paternal education status on measles vaccination update in nations with the highest numbers of missed measles vaccinations.

The relative increases in female education may account, in part, for the substantial improvements in measles immunisation coverage. With global gender parity in education status now virtually assured based on MDG trends, paternal education is likely to play a critical role in efforts to improve childhood immunisation. This is particularly the case in all nations with the highest numbers of unimmunised children, where patrilineal norms predominate. For example, a study from northern Nigeria based on a 2007 dataset revealed that children whose fathers approve of BCG immunization are almost three times as likely to be immunized as those whose fathers disapprove [16]. Similar trends are presumed for other nations with large numbers of unimmunised children.

Addressing paternal encumbrances to measles vaccination entails interventions at three levels. First, improvement in male educational levels, particularly in rural regions of developing nations where the labour input of children exceeds potential gains from poor-quality secular education. MDGs are based on male educational level as a reference. However, as male educational levels are currently sub-optimal in many developing nations, averaging 77% for proportion progressing through secondary school in the six nations studied, according to latest World Bank estimates [17], achieving gender parity in formal education will still leave a large proportion of individuals illiterate, both male and female. Greater investments in creating educational opportunities for children in developing nations, and removal of encumbrances such as excessive school fees are urgently required [18]. Second, reduce educational, occupational, cultural, religious, socio-economic and political encumbrances to gender equality, such as to enhance the opinions of mothers on issues of child health within families. Third, adequate health communication strategies on the benefits of vaccination should be designed to appeal to both fathers and mothers. Community and religious leaders should be encouraged to advocate in favour of vaccination. It is acknowledged that removing the encumbrances on paternal education in nations performing poorly with regards to measles vaccination will not result in significant progress unless vaccination-related issues are concurrently addressed at other levels [6].

We have identified three limitations in this study. First, measles vaccination data relied heavily on mother’s recall, which is subject to bias. Maternal recall however, is considered a valid measure of child vaccination coverage in the absence of vaccination records in developing countries [19]. It is likely that many children in the sampled countries did not have a completed vaccination card, for example, only 21% of children the Indonesian sample produced a complete vaccination card. Second, the effects of contextual factors such as social influence and social learning on the decision to have children vaccinated were not considered [20]. It is possible that both parent education levels and the level of education of those in the community may influence the likelihood that children are vaccinated against measles. Finally, factors relating to the availability and accessibility of primary health infrastructure were not considered due to the unavailability of data.

Despite these limitations, this study shows that it is important to appreciate the role of the key decision maker in the household in nations with the lowest measles immunisation coverage- the father. This would involve the implementation of interventions which encourage fathers to work cooperatively with mothers and other relatives to vaccinate children. This is critical in making further progress in improving measles vaccination uptake and facilitating the goal of measles eradication by 2020 [16].

## **Conclusions**

Inter-personal factors (i.e. parental and household factors) are important determinants of uptake of measles and other childhood vaccinations [6]. Commendable public health efforts have been devoted to improving maternal education as part of a comprehensive child health development strategy, based on studies showing that parental education has a stronger impact on child morbidity and mortality compared with household income. However, most of these studies have selectively emphasised the impact of maternal education [21-23]. Studies which understate the significance of paternal education in optimal child development appear not to have taken “assortative matching” into account [15]. We have shown in our study that, in relation to one-off child health decisions such as childhood vaccinations, paternal education status independently impacts on child vaccination uptake, and may be more significant than the impact of maternal education status in countries with high measles burden and significant missed vaccinations. Marks et al. also found a higher impact of increasing paternal education compared with increasing maternal education on optimising childhood vaccination uptake in Ohio, United States, using 1975 data [12]. As female educational achievements continue to equal, and in some nations outstrip, average educational achievements of males globally, greater support is required to enhance male educational levels, not least as a pathway to optimising children’s development though better utilisation of child health services.

## **Competing interests**

We declare that there is no financial or non-financial conflict of interest in relation to this article.

## **Authors’ contributions**

All authors contributed equally to the conceptualisation, data analysis, literature review and writing of this manuscript.

## Pre-publication history

The pre-publication history for this paper can be accessed here:

http://www.biomedcentral.com/1471-2458/12/336/prepub
